# Commentary: The “hat trick” of cardiac surgery

**DOI:** 10.1016/j.xjtc.2021.07.011

**Published:** 2021-07-21

**Authors:** David L. Joyce

**Affiliations:** Department of Surgery, Medical College of Wisconsin, Hub for Collaborative Medicine, Milwaukee, Wis

**Figure:**
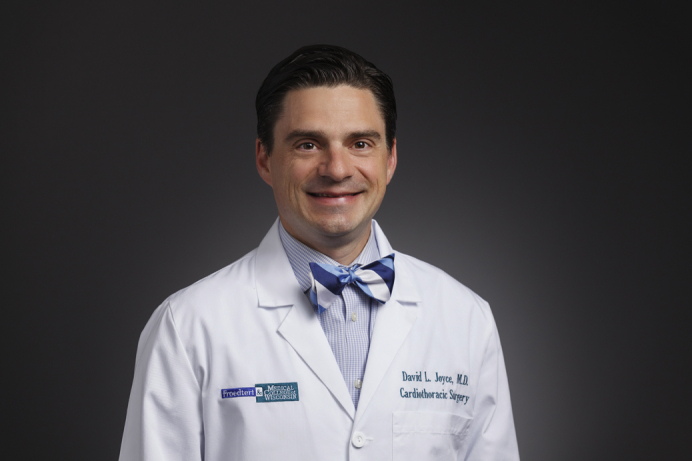
David L. Joyce, MD, MBA

**Central Message**

Complex tricuspid valve repair techniques that avoid the use of prosthetic material carry the potential for improved long-term outcomes.

See Article page 293.

Consider the “pull the goalie” scenario confronted by this Cliniques Universitaires Saint-Luc team in delivering such a spectacular victory for this unfortunate young man.^1^ First, the patient would have to withstand the risks of septic emboli, bacteremia, and large persistent vegetations on the tricuspid valve heading into the operating room since the urgency of the procedure did not allow for optimization. Second, in what can only be described as a game of microns, the vegetations would have to be completely removed while preserving enough free margin of the anterior leaflet to allow for adequate reconstruction. The third and most formidable obstacle would occur at hospital discharge—the very moment when victory is all but assured for many cardiac operations. Substance use disorder has been shown to be the strongest predictor of mortality in these types of cases,^2^ and a perfect repair could be easily nullified with the insertion of even one infected needle. While there are no medical idioms to characterize the significance of going 3 for 3 against these types of challenges, in the world of hockey (or cricket, depending on your continent) this case would enter the record books as a “hat trick.”

But even as we admire the masterful techniques on display in the accompanying video, we are left wondering if further innovation in the management of tricuspid valve endocarditis could swing the odds so heavily in favor of long-term survival as to make these sorts of clever solutions seem unnecessary. To that end, a number of technology solutions are already on the horizon. Traditional indications for urgent surgery include large persistent vegetations, right heart failure in the setting of severe tricuspid regurgitation, and recurrent pulmonary emboli.^3^ However, one wonders if an ounce of optimization might be worth a pound of postoperative recovery in avoiding the “protracted” course described in this case. In our center, we have established a multidisciplinary approach that leverages the benefits of percutaneous vacuum-assisted devices and mechanical circulatory support to improve right ventricular hemodynamics while sterilizing the bloodstream over days to weeks. Despite the superb technical result achieved in this case, the reproducibility of these types of complex repair techniques have yet to be determined. In an effort to develop a “one-size-fits-all” procedure, a novel extracellular matrix cylinder reconstruction technique has been adopted by a number of centers with promising early outcomes.^4^ The effectiveness of this approach is currently under investigation as part of a proof of principle trial.^5^ While surgeons are unlikely to solve the recidivism problem on their own, a growing body of evidence in Europe suggests that embracing a multidisciplinary approach to endocarditis leads to improvements in morbidity and mortality.^6^^,^^7^ This strategy has worked well in the heart failure population, where substance abuse often precludes candidacy for transplantation. While the management of tricuspid valve endocarditis is without question a team sport, the implementation of techniques like the one described in this case report will go a long way toward achieving long-term success.
